# Supplementary material for: Free-standing ultrathin silicon wafers and solar cells through edges reinforcement
Source: Nat Commun. 2024 May 7;15:3843. doi: 10.1038/s41467-024-48290-5 (PMC11076549; doi:10.1038/s41467-024-48290-5)
Supplement: Supplementary file 1 — Supplementary Information [file 41467_2024_48290_MOESM1_ESM.pdf]

Supplementary information

Free-standing ultrathin silicon wafers and solar cells through edges reinforcement

Taojian Wu[1,#], Zhaolang Liu[2,#], Hao Lin[2,3]*, Pingqi Gao[2,3,4]*, Wenzhong Shen[1]*

[1] Institute of Solar Energy, Key Laboratory of Artificial Structures and Quantum Control (Ministry of Education), School of Physics and Astronomy, Shanghai Jiao Tong University, 800 Dong Chuan Road, Shanghai 200240, China

[2] School of Materials, Shenzhen Campus of Sun Yat-sen University, No. 66, Gongchang Road, Shenzhen, Guangdong 518107, China

[3] Institute for Solar Energy Systems, State Key Laboratory of Optoelectronic Materials and Technologies, Sun Yat-sen University, Guangzhou 510275, China

[4] Jiangsu Collaborative Innovation Center of Photovoltaic Science and Engineering, Changzhou University, Changzhou, 213164, China

[#] These authors contributed equally.

* Corresponding author. linh229@mail.sysu.edu.cn (H. L.); gaopq3@mail.sysu.edu.cn (P. G.); wzshen@sjtu.edu.cn (W. S.).

Supplementary Figures

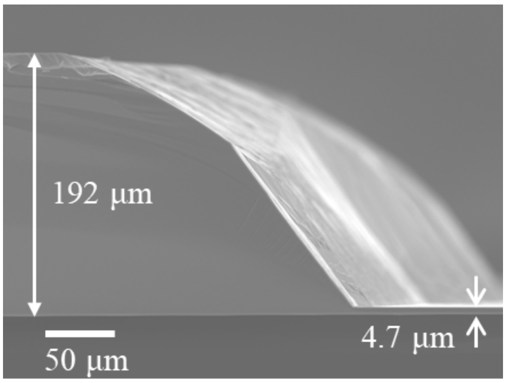

Supplementary Figure 1. Boundary information of TSRR structure. SEM image about the boundary of the reinforced ring and the central thin silicon region of TSRR structure.

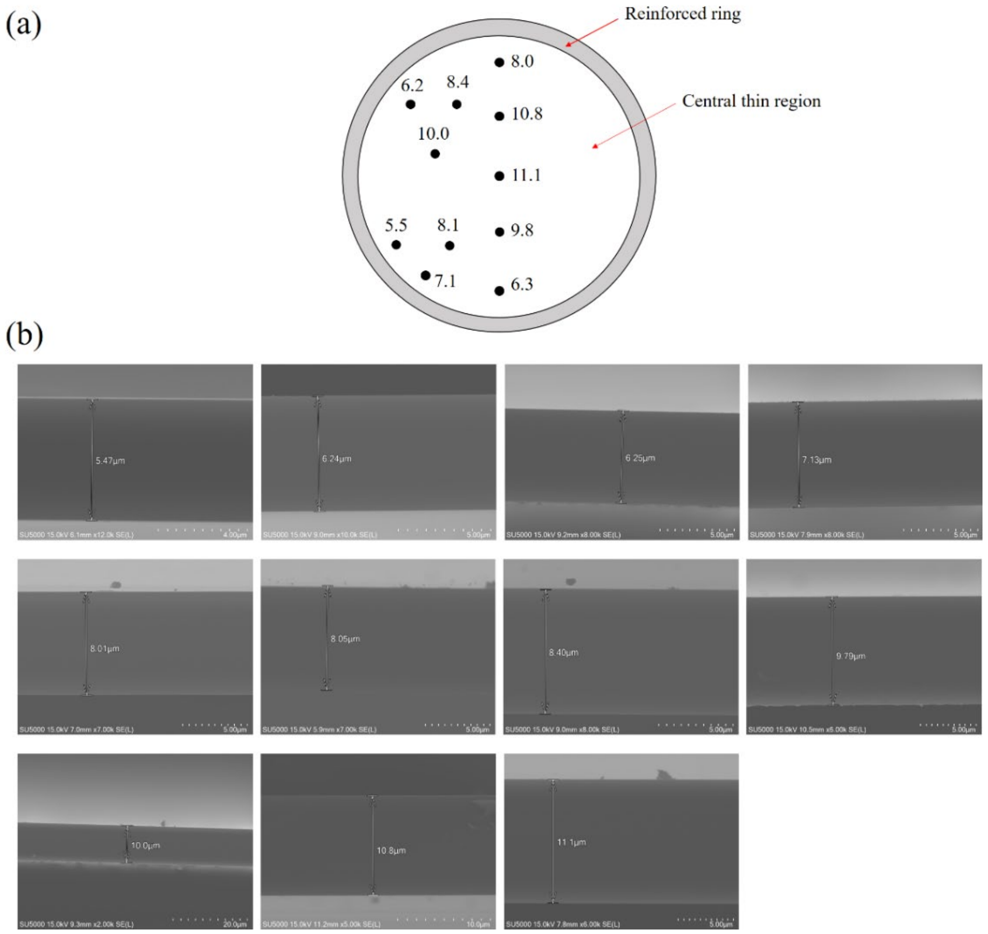

Supplementary Figure 2. Thickness uniformity of TSRR structure. (a) The quantitative thickness uniformity measurement results in a 4-inch wafer with TSRR structure and (b) the corresponding SEM images (in order from thin to thick) of the cross section for each point in (a). 11 points were measured and their thicknesses are from 5.5 to 11.1 μm, i.e., the total thickness variation (TTV) is within 6 μm.

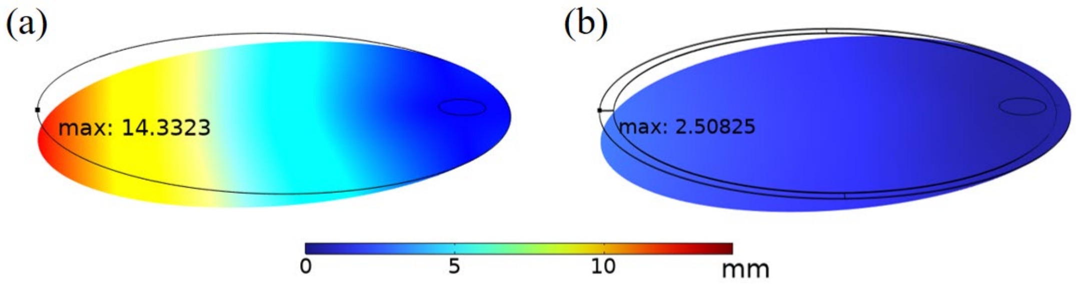

Supplementary Figure 3. Simulated displacement by COMSOL. Displacement of thin silicon wafer with (a) ATS and (b) TSRR structures under the effect of gravity (with a fixed position).

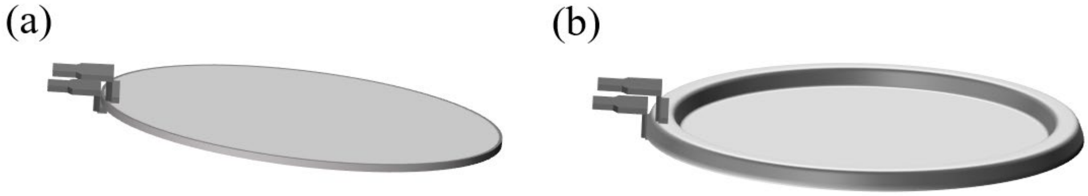

Supplementary Figure 4. Practical situations when handling or transferring. The schematic diagram when handling or transferring the (a) ATS and (b) TSRR structures.

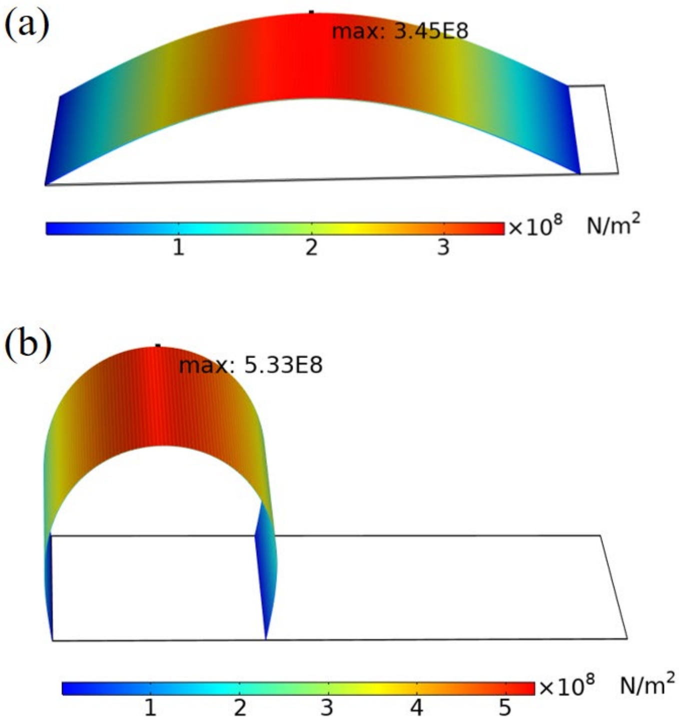

Supplementary Figure 5. Fracture simulations and experiments for 30 mm long crystalline silicon samples. (a) The simulated Von Mises stress profile (left) and experiment (right) of 77-μm thick silicon with ATS structure when the critical displacement is 2 mm. (b) The simulated Von Mises stress profile (left) and experiment (right) of 32-μm thick silicon with ATS structure when the critical displacement is 19 mm. According to the simulations and experiments, we can conclude that the strength of crystalline silicon is 345~533 × 10^6 N/m^2 = 345~533 MPa.

中国认可
国际互认
检测
TESTING
CNAS L8490

Test and Calibration Center of New Energy Device and Module,
Shanghai Institute of Microsystem and Information Technology,
Chinese Academy of Sciences (SIMIT)

Measurement Report

Report No. 24TR040701

**Client Name**	Shanghai Jiao Tong University
**Client Address**	800 Dong Chuan Road, Shanghai, China
**Sample**	Thin crystalline silicon solar cell
**Manufacturer**	Shanghai Jiao Tong University, Sun Yat-sen University
**Measurement Date**	7th April, 2024

**Performed by:**	Qiang Shi Qiang Shi	**Date:** 07/04/2024
**Reviewed by:**	Wenjie Zhao Wenjie Zhao	**Date:** 07/04/2024
**Approved by:**	Yucheng Liu Yucheng Liu	**Date:** 07/04/2024

Address: No.235 Chengbei Road, Jiading, Shanghai **Post Code:**201800
E-mail: solarcell@mail.sim.ac.cn **Tel:** +86-021-69976905

The measurement report without signature and seal are not valid.
This report shall not be reproduced, except in full, without the approval of SIMIT.

Report No. 24TR040701

Sample Information

Sample Type	Thin crystalline silicon solar cell, IBC
Serial No.	05-2-7
Lab Internal No.	24040701-1#
Measurement Item	I-V characteristic
Measurement Environment	24.4±2.0°C, 40.3±5.0%R.H

Measurement of I-V characteristic

**Reference cell**	PVM1121
**Reference cell Type**	mono-Si, WPVS, calibrated by NREL (Certificate No. ISO 2098)
**Calibration Value/Date of Calibration for Reference cell**	143.95mA/ Feb. 2024
**Measurement Conditions**	Standard Test Condition (STC):<br>Spectral Distribution: AM1.5 according to IEC 60904-3 Ed.3,<br>Irradiance: 1000±50W/m^2, Temperature: 25±2°C
**Measurement Equipment/ Date of Calibration**	AAA Steady State Solar Simulator (YSS-T155-2M) / July.2023<br>IV test system (ADCMT 6246) / June. 2023<br>Measuring Microscope (MF-B2017C) / July.2023<br>SR Measurement system (CEP-25ML-CAS) / April.2023
**Measurement Method**	I-V Measurement:<br>Logarithmic sweep in reverse direction (Voc to Isc) during one flash based on IEC 60904-1:2020;<br>Spectral Mismatch factor was calculated according to IEC 60904-7 and I-V correction according to IEC 60891.
**Measurement Uncertainty**	Area: 1.0%(k=2); Isc: 2.0%(k=2); Voc: 1.0%(k=2);<br>Pmax: 2.4%(k=2); Eff: 2.5%(k=2)

SIMIT

Report No. 24TR040701

====**Measurement Results** ====

Area [cm^2]	Isc [mA]	Voc [V]	Pmax [mW]	FF [%]	Eff [%]
1.0070	36.517	0.7577	20.186	72.96	20.05

- Spectral Mismatch Factor SMM=0.9958.
- Aperture area defined by a thin black mask was measured by a measuring microscope.
- Test results listed in this measurement report refer exclusively to the mentioned measured samples.
- The results apply only at the time of the test, and do not imply future performance.

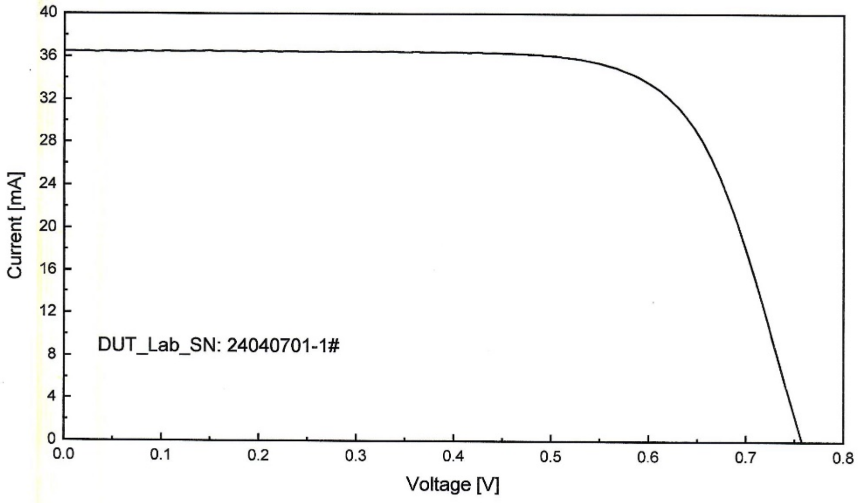

Fig.1 I-V curve of the measured sample

-------**End of Report**-------

3 / 3

Supplementary Figure 6. Third-party certification. Independent certification by Shanghai Institute of Microsystem and Information Technology (SIMIT) of the TSRR solar cell. Published with permission of SIMIT.

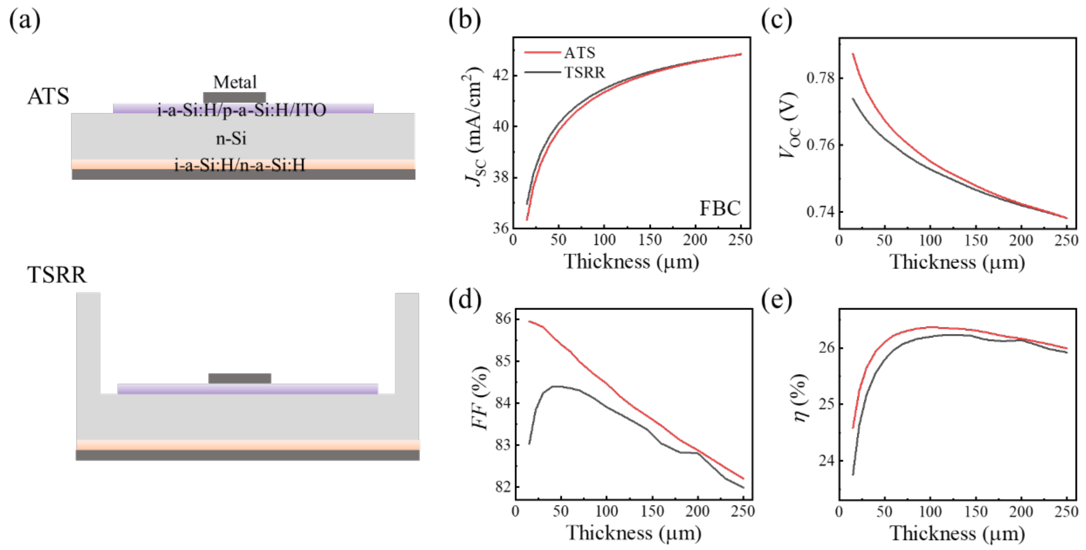

Supplementary Figure 7. Comparison of the optoelectrical performance of the ATS and TSRR solar cells with FBC structure. (a) The simulated ATS and TSRR solar cells with front and back contacts (FBC). (b) J_{SC}, (c) V_{OC}, (d) *FF*, and (e) η as a function of thickness for ATS and TSRR solar cells. Please see supplementary Discussion 1 for a more detailed discussion. Source data are provided as a Source Data file.

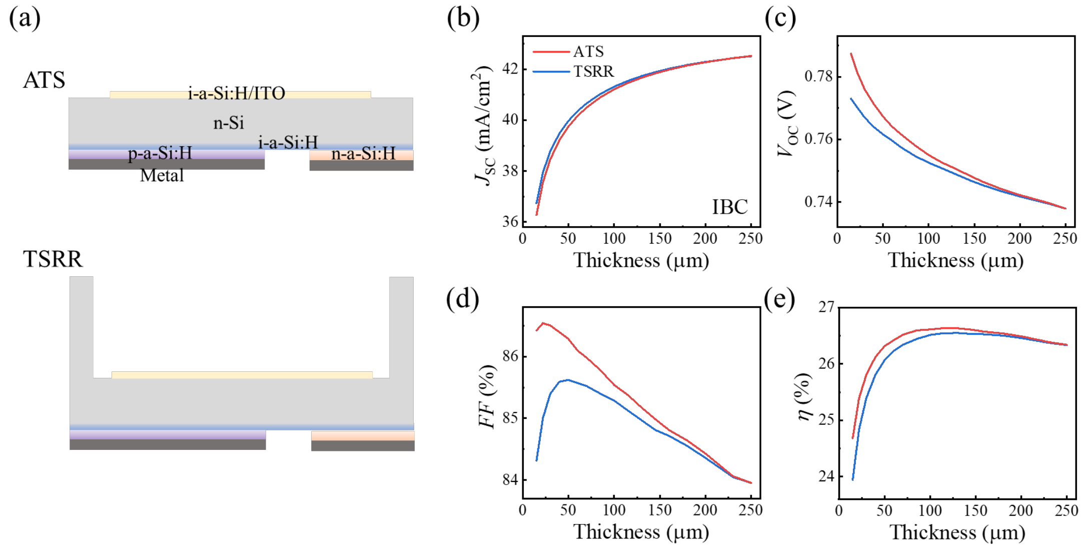

Supplementary Figure 8. Comparison of the optoelectrical performance of the ATS and TSRR solar cells with IBC structure. (a) The simulated ATS and TSRR solar cells with interdigitated back contacts (IBC). (b) J_{SC}, (c) V_{OC}, (d) *FF*, and (e) η as a function of thickness for ATS and TSRR solar cells. Source data are provided as a Source Data file.

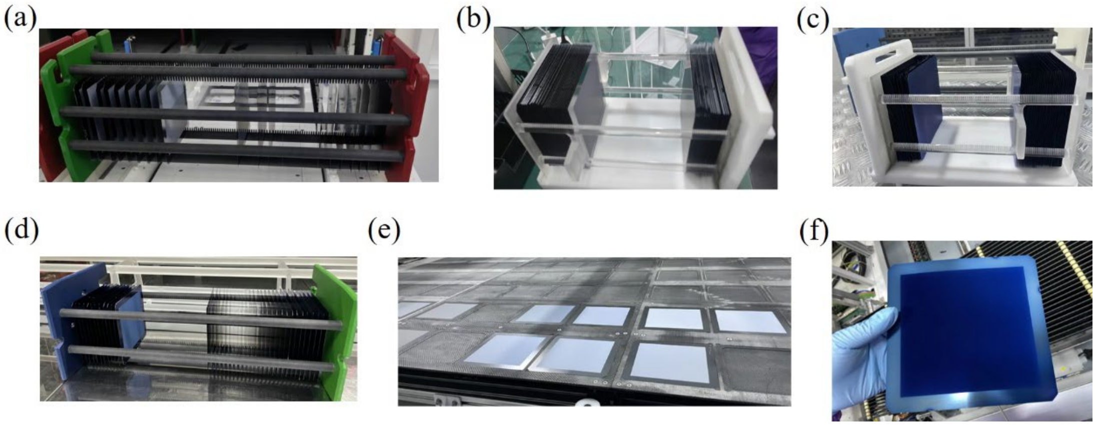

Supplementary Figure 9. Industrial compatibility validation of TSRR structure. Pictures of samples after step (a) 1, (b) 2, (c) 3, (d) 6, (e) 9 and (f) 11. The process steps are: (1) previous RCA cleaning, (2) front-side boron diffusion, (3) back-side thermal SiO_2 at 1050 ℃, (4) single-side SiO_2 removal, (5) alkali polishing, (6) p^+ doped poly-Si deposition at 430 ℃ and annealing at 900 ℃, (7) front-side phosphosilicate glass (PSG) removal, (8) RCA cleaning, (9) Al_2O_3 deposition by atomic layer deposition (ALD) for surface passivation, (10) front-side and (11) back-side PECVD SiNx at 520 ℃ as antireflection layer.

Supplementary Discussion

Supplementary Discussion 1. Comparison of the optoelectrical performance of the ATS and TSRR solar cells. As shown in Supplementary Figure 7a, it is the simulated optoelectrical performance of the ATS and TSRR solar cells with FBC structure. The simulation parameters can be found in Table 4 and the thickness of the reinforced ring and the Ratio for TSRR structure are 250 μm and 5%, respectively. Let's look into Supplementary Figure 7b, when the thickness is 250 μm, the J_{SC} of ATS and TSRR structures are the same since the reinforced ring of TSRR structure is also 250 μm. J_{SC} decreases as the thickness decreases because thinner silicon body absorbs less light. Besides, the J_{SC} of TSRR structure is always larger than that of ATS structure due to the presence of thicker reinforced ring in TSRR structure. Next, let's move on to Supplementary Figure 7c. V_{OC} increases as the thickness decreases due to reduced bulk recombination. Similarly to the J_{SC}, the V_{OC} of TSRR structure is always smaller than that of ATS structure due to more bulk recombination in the thick reinforced ring of TSRR structure. As for *FF*, when the thickness is 250 μm, there is a 0.2% difference between the two structures attributed to the difference in mesh settings in the simulation (the results shown in Supplementary Figure 8 is more accurate because that simulation is based on the contacts being all on the backside), as indicated in Supplementary Figure 7d. As thickness decreases, *FF* increases for ATS structure since the longitudinal distance required for carrier transport to the electrodes decreases. (Note that if the thickness is particularly low (<10 μm), it will cause the *FF* to decrease, because the resistance of lateral transport of carriers to the front electrodes increases at that point). While for TSRR structure, (as mentioned in the article) the photogenerated carriers in the reinforced ring region need to transport to the metal electrodes to be collected, which affect the *FF*, leading to a lower *FF* for the TSRR structure than the ATS structure, and *FF* begins to decrease at around 50 μm. The final η for both structures are demonstrated in Supplementary Figure 7e.
