# Supplementary material for: Free-standing ultrathin silicon wafers and solar cells through edges reinforcement
Source: Nat Commun. 2024 May 7;15:3843. doi: 10.1038/s41467-024-48290-5 (PMC11076549; doi:10.1038/s41467-024-48290-5)
Supplement: Supplementary file 3 — Description of Additional Supplementary Files [file 41467_2024_48290_MOESM3_ESM.pdf]

File name: Supplementary Movie 1

Description: Comparison of the behaviour of ATS and TSRR structures during the alkaline solution etching wet process.
