# Supplementary material for: Free-standing ultrathin silicon wafers and solar cells through edges reinforcement
Source: Nat Commun. 2024 May 7;15:3843. doi: 10.1038/s41467-024-48290-5 (PMC11076549; doi:10.1038/s41467-024-48290-5)
Supplement: Supplementary file 5 — Solar Cells Reporting Summary [file 41467_2024_48290_MOESM5_ESM.pdf]

nature portfolio

Corresponding author(s): Hao Lin, Pingqi Gao, Wenzhong Shen

Solar Cells Reporting Summary

Nature Portfolio wishes to improve the reproducibility of the work that we publish. This form is intended for publication with all accepted papers reporting the characterization of photovoltaic devices and provides structure for consistency and transparency in reporting. Some list items might not apply to an individual manuscript, but all fields must be completed for clarity.

For further information on Nature Research policies, including our data availability policy, see Authors & Referees.

Experimental design

Please check the following details are reported in the manuscript, and provide a brief description or explanation where applicable.

1. Dimensions

Area of the tested solar cells

- [x] Yes — The active area is 1.007 cm2.
- [ ] No — *Explain why this information is not reported/not relevant.*

Method used to determine the device area

- [x] Yes — A thin black mask is used to define the cell area, and the area of the mask is measured by a measuring microscope. This is stated in the certification report.
- [ ] No — *Explain why this information is not reported/not relevant.*

2. Current-voltage characterization

Current density-voltage (J-V) plots in both forward and backward direction

- [x] Yes — JV curve in forward direction is given in the manuscript and JV curve in backward direction are performed and shown in the certification report.
- [ ] No

Voltage scan conditions

- [x] Yes — Forward direction from: -0.4V to 1.1V, with 300 data points.
- [ ] No — *Explain why this information is not reported/not relevant.*

Test environment

- [x] Yes — Standard testing conditions (AM1.5G, 1000 W/m², 25°C) . This is stated in the certification report.
- [ ] No — *Explain why this information is not reported/not relevant.*

Protocol for preconditioning of the device before its characterization

- [ ] Yes — *Provide a description of the protocol.*
- [x] No — No preconditioning was used.

Stability of the J-V characteristic

- [ ] Yes — *Provide a description of the method used. The stability of the J-V characteristic can be verified with time evolution of the maximum power point or with the photocurrent at maximum power point; see ref. 5 for details.*
- [x] No — Stability of the J-V curves was not performed.

3. Hysteresis or any other unusual behaviour

Description of the unusual behaviour observed during the characterization

- [ ] Yes — *Provide a description of hysteresis or any other unusual behaviour observed during the characterization.*
- [x] No — The Silicon solar cells did not exhibit hysteresis under standard conditions.

Related experimental data

- [ ] Yes — *Provide a description of the related experimental data.*
- [x] No — The Silicon solar cells did not exhibit hysteresis under standard conditions.

4. Efficiency

External quantum efficiency (EQE) or incident photons to current efficiency (IPCE)

- [x] Yes — The EQE is presented in the Figure 3.
- [ ] No — *Explain why this information is not reported/not relevant.*

A comparison between the integrated response under the standard reference spectrum and the response measure under the simulator

- [x] Yes — The integrated short-circuit current from the EQE spectrum is the same as that measured under the solar simulator, as shown in Figure 3.
- [ ] No — *Explain why this information is not reported/not relevant.*

For tandem solar cells, the bias illumination and bias voltage used for each subcell

- [ ] Yes
- [x] No

Provide a description of the measurement conditions.

We do not report on tandem solar cells.

5. Calibration

Light source and reference cell or sensor used for the characterization

- [x] Yes
- [ ] No

The light source is a AAA Sun Simulator and the reference cell is a mono-silicon and WPVS cell. This is stated in the certification report.

Explain why this information is not reported/not relevant.

Confirmation that the reference cell was calibrated and certified

- [x] Yes
- [ ] No

The reference WPVS cell is calibrated by NREL. This is stated in the certification report.

Explain why this information is not reported/not relevant.

Calculation of spectral mismatch between the reference cell and the devices under test

- [x] Yes
- [ ] No

Spectral mismatch factor was calculated according to IEC 60904-7 and the mismatch factor was 0.9958. This is stated in the certification report.

Explain why this information is not reported/not relevant.

6. Mask/aperture

Size of the mask/aperture used during testing

- [x] Yes
- [ ] No

The area of the mask is 1.007 cm2.

Explain why this information is not reported/not relevant.

Variation of the measured short-circuit current density with the mask/aperture area

- [ ] Yes
- [x] No

Report the difference in the short-circuit current density values measured with the mask and aperture area.

We did not perform the test that changing the mask area.

7. Performance certification

Identity of the independent certification laboratory that confirmed the photovoltaic performance

- [x] Yes
- [ ] No

Confirmed by Shanghai Institute of Microsystem and Information Technology (SIMIT).

Explain why this information is not reported/not relevant.

A copy of any certificate(s)

- [x] Yes
- [ ] No

It can be found in Supplementary Fig. 6.

Explain why this information is not reported/not relevant.

8. Statistics

Number of solar cells tested

- [x] Yes
- [ ] No

We prepare 4 solar cells at a time which go through the same process steps. The certified and reported performance is from the champion cell.

Explain why this information is not reported/not relevant.

Statistical analysis of the device performance

- [ ] Yes
- [x] No

State where this information can be found in the text.

It only shows the performance of the champion solar cells.

9. Long-term stability analysis

Type of analysis, bias conditions and environmental conditions

- [ ] Yes
- [x] No

Provide a description of the type of analysis, bias conditions and environmental conditions (e.g. illumination type, temperature, atmosphere humidity, encapsulation method, preconditioning temperature, bias) for each long-term stability analysis carried out; see ref. 7 and 8 for details.

Stability analysis was not performed in this manuscript.
